# Dr. Maclean, on the Digitalis Purpurea

**Published:** 1799-09

**Authors:** L. Maclean

**Affiliations:** Sudbury, Suffolk


### *To the Editors of the Medical and Phyfical Journal.*

GENTLEMEN,

IF you think the following remarks on Digitalis Purpurea worthy a place in the Medical and Phyfical Journal, you are at liberty to difpofe of them as you may think proper, and I have the honour to be,

Gentlemen,

Your obedient fervant,

L. MACLEAN, M. D.

SUDBURY, SUFFOLK,
*Aug.* 4, 1799.

Among the many valuable facts and obfervations with which the Medical and Phyfical Journal abounds, thofe which relate to the Digitalis Purpurea are not the leaft interefting. It is with pleafure I obferve the real properties of this long-neglected vegetable begin at length to be known in different parts of the world. It has long been a favourite medicine with me, and this partiality is founded on the fulleft evidence of its extraordinary efficacy, in fome of the moft formidable difeafes incident to mankind.

When I fixed my refidence in this country (nine years fince the perufal of Dr. WITHERING's excellent work), what I had feen and heard of its efficacy in dropfy impreffed on my mind fo favourable a bias towards it, that I was anxious for opportunities of putting its virtues to the teft. The refult has been extremely fatisfactory. After feveral difappointments, arifing from the quality of the preparations at firft ufed, my fuccefs for upwards of five years has exceeded my moft fanguine expectations.

In the courfe of my experience, many circumftances attracted my notice which I was before unacquainted with, and which induced me to pay more than ordinary attention to its peculiar effects on the living fibre. Thefe I began at an early period to minute down in my Journal, intending to commit them to the prefs as foon as they appeared fufficiently interefting for public infpection. But the ingenious communications of Doctors FOWLER and DRAKE, the comments and additional reports of the learned Editors of the valuable works in which they are inferted, lead me to deviate in fome degree from my original defign, and to introduce, without farther delay, to public view, the general refults of my experience of the fox-glove in confumption, and a few other diforders, felected from the more detailed cafes and obfervations recorded in my Note-book.

I am aware my experience has as yet been too limited to enable me to fpeak with precifion of the real extent of its powers in confumption; yet when I hear men profoundly verfed in medical fcience mention it in terms of the moft extravagant commendation, ftamping upon it a character little fhort of infallibility, while others ſpeak of it as a deſtructive poiſon, that muſt be reſorted to with the greateſt circumſpection, and many are totally ignorant of its real properties, doſes, and mode of exhibition, I truſt I may be pardoned thus prematurely intruding my obſervations on the public.

I am perpetually hearing that Dr. Drake has diſcovered a ſpecific for conſumption; and although this is to be attributed to the well-meant, but miſtaken zeal of his friends, yet the concluſions * deduced from the ſucceſsful reſults of two caſes, are obviouſly calculated to inſpire the fulleſt confidence in it;—a confidence which the bark in intermittents, or mercury in lues, ſcarcely warrants.

The reſult of the firſt few trials † made by Dr. Beddoes was not very encouraging; but his ſubſequent experience ‡ of it has been ſo flattering, that he is induced to hold out the moſt ſanguine expectations of it.

Dr. Fowler's ſucceſs § has been great alſo; yet, like a true philoſopher, a cautious and experienced practitioner, he has contented himſelf with merely detailing the caſes, leaving the public at liberty to draw their own concluſions, withholding *his* until farther trials might enable him to ſpeak more decidedly of the real powers of the medicine.

Dr. Beddoes's name is familiar wherever the ſciences are cultivated, his capacious mind having embraced every branch of them; and his zeal in making his labours and diſcoveries more eſpecially ſubſervient to the health and comfort of his fellow-creatures, is highly meritorious, and lays the public under weighty obligations to him.

Dr. Fowler likewiſe ſtands deſervedly high as a man of ſcience, and a judicious practitioner.

Dr. Drake, too, I know, from perſonal acquaintance, to be a man of character and of talents.

Hence the high encomiums beſtowed upon the fox-glove in conſumption, by ſuch reſpectable authorities, muſt neceſſarily have conſiderable influence, not only within the immediate ſphere of their practice, but with the medical world at large.

But whether ſubſequent trials be attended with effects equally happy, the public will ſpeedily be in poſſeſſion of evidence ſufficient to enable them to decide

* "Medical Contributions," p. 486. "Medical and Physical Journal," p. 291.

† "Medical Contributions," p 521. "Medical and Physical Journal," p. 293.

‡ "Essay on Consumption," p. 270. "Medical and Physical Journal." p. 384.

§ "Medical Contributions," p. 500. "Medical and Physical Journal," p. 291.

decide. If even it fall ſhort by ſome degrees of performing what is promiſed from it, there will, as Dr. Beddoes emphatically expreſſes it, "be a cauſe for national rejoicing, greater and more univerſal than has before occurred *."

My own experience in upwards of twenty caſes will not ſuffer me to ſpeak in ſuch high terms of it. I have certainly found it a moſt valuable antiphthiſical remedy, and although I truſt it will be found by others the moſt efficacious that has hitherto been reſorted to, yet its powers are limited, even in the early ſtages.

I was firſt encouraged to preſcribe it in pulmonary conſumption, from the happy effects experienced from it in the chronic cough, dyſpnœa, diſtreſſing pain and ſtricture acroſs the cheſt frequently attending hydrothorax, and ſometimes remaining after the ſerous fluid was evacuated. It was exhibited under the moſt favourable impreſſions, and when my mind was not influenced by theory, and no circumſtances tending to inſure its ſucceſs were overlooked. The caſes were not ſelected for my purpoſe, but ſuch as chanced to fall under my care in the regular routine of practice. Some of theſe were of the true genuine conſumptive kind (by which I mean, that which ariſes under peculiar circumſtances of conſtitution, accurately delineated by ſeveral authors; is in general hereditary and connected with a ſcrophulous habit; appearing from ſixteen to thirty, but more eſpecially under twenty years of age, and ſweeping away whole families, particularly females, no leſs diſtinguiſhed for high mental endowments, than by beauty and perſonal accompliſhments, without even being checked in its fatal career); others ſupervened to neglected or improperly treated catarrhal affections; and pneumonic inflammation, at various ages and apparently unconnected with this peculiar habit, and theſe were at various ſtages, and conſequently of different degrees of violence.

The ſucceſs of the fox-glove, as is the caſe with every remedy, was proportioned to its early exhibition. Inſtances continually occur to practitioners with all the characters of phthiſis, but without the exiſtence of tubercles; there is inceſſant cough, ſometimes with copious expectoration of a yellow-matter, ſometimes without; the hectic fever, with morning or evening exacerbations, or both, and night ſweats, are ſtrongly marked. Such inſtances are more common among huſbandmen than others, and ariſe from their continuing their labour in the fields, expoſed to all the viciſſitudes of temperature, under violent colds or catarrhal affections; but if the phthiſical habit does not exiſt, they frequently yield to a particular treatment. In theſe the

* "Essay on Consumption," p. 271. et seq.

the fox-glove will be found an excellent remedy. Many cafes of chlorofis are of this defcription: there is cough, generally dry; pain of the fide, chillinefs, fucceeded by heat, intenfe thirft, and fometimes night-fweats. Many fuch, that had been pronounced confirmed phthifis by a noted con-fumption-doctor, have been reftored to health under my care, by means of fteel, myrrh, bark, with faline medicines occafionally, and fuitable regimen. The true, genuine, fatal difeafe, however, is fometimes ufhered in, or attended with chlorotic fymptoms.

But although the fox-glove is chiefly to be relied upon in fuch cafes and in the early ftages, yet I was fortunate in refcuing a few from death, after all other means failed. Two of thefe, Mr. Brewster, of Melford, aged thirty-two, and Mifs L. Canham, of Edwardfton, aged fifteen years, I attended with Mr. Edwards, of Melford, and Mr. Wynne, of Boxford; to whofe kindnefs in attending to the plan I recommended, and in preferving minutes of the cafes, I feel much indebted. The tincture was the prepara-tion ufed. Mr. Brewfter was kept for fome weeks under its full influence; Mifs Canham not fo long. Four of Mr. Brewfter's family, and as many of Mifs Canham's family died of confumption.

Some of my patients for a time, "advanced," to ufe Dr. Beddoes's ex-preffion, "with a firm pace towards recovery;" but the pace became gra-dually lefs and lefs firm, until they ceafed at length to make any progrefs; and after remaining ftationary for a certain time, all the unpromifing fymp-toms returned in the fame gradual manner they had receded, and the diforder terminated its fatal career in defiance of all my efforts. In a few, however, when by the aid of the digitalis, the hectic fymptoms were fubdued, and only weaknefs remained, tonics completed the cure.

In the cafes of two young ladies (fifters) in this neighbourhood, the one feventeen, the other fifteen years of age, labouring under confirmed phthifis, apparently much more favourable than thofe I have related of its fuccefs, it completely failed. It was firft given in powder to one; afterwards in tinc-ture to both, in combination and alone; introduced gradually and increafed to the fulleft extent the habit was capable of bearing; or at once in large dofes. It was repeatedly difcontinued, fometimes by degrees, fometimes fuddenly, for fome time, and then had recourfe to again, but in vain. The fymptoms were evidently for a time not only relieved, but checked in their progrefs in both. The elder for feveral nights could not fleep without a full dofe (gtt. xxx.) at bed time. This for a time infured a good night, but it ultimately ceafed to do any good; and the elder is finking under the difeafe, while the fituation of the younger is extremely precarious, and all medicine has has been abandoned as ufelefs. In one the diforder had been checked more than once before, but the means failed now; and an elder fifter fell a facrifice a few years before, after the progrefs had been fufpended during the fummer months three different feafons.

Mr. Harrold, of Nayland, an intelligent practitioner, on whofe fidelity and accuracy in attending to its effects, in the intervals of my vifits, the fulleft reliance may be placed, was equally defirous with me in giving the digitalis a trial, and equally interefted in its fuccefs. Hence it may be fuppofed to have had a fair chance.

On the whole then, the fox-glove will be found a valuable remedy in confumption. It will fometimes cure when the moft approved remedies fail. When of itfelf it is infufficient to fubdue the difeafe, it will prove a valuable auxiliary to other means. It has always, with me, quieted and foothed the fufferings of the patient more or lefs; and where it ultimately failed, it lengthened the duration of life, and fmoothed the avenues to death.

This is all I apprehend it will be found capable of performing; but this is doing a great deal. Thofe who expect wonders from it, or that it will *in general* cure confumption, will be difappointed, I lament it is not poffible for me at prefent to form a correct eftimate of the proportion of fuccefsful to the unfuccefsful cafes, becaufe I fee the moft ferious evils begin already to refult from its not anfwering the high expectations that have been raifed.

A phyfician of eminence and extenfive practice in this county, from the ftrong recommendation of Dr. Drake, was readily induced to give trial to it in three cafes of phthifis, and he affured me it had fair play; but from its failure in all, he can fcarcely be prevailed upon to have recourfe to it again. In one of thefe the difeafe was afterwards fufpended for feveral months, by change of air and fuitable regimen, under the direction of a phyfician of eminence in town, who reprobated in pointed terms the exhibition of fox-glove, and I hear, declared the cafe not confumption. But the lady has lately fallen a victim to the moft confirmed and diftinctly marked hectic that ever occurred.

Others begin to lofe their confidence in it from fimilar failures, whereas, had it been brought forward with its true character ftamped upon it, this would not be the cafe. Digitalis will be found highly beneficial in afthmatic affections, coughs of every kind, dyfpnœa, and other chronic affections of the cheft and lungs. It promifes to prove ufeful in hooping-cough; and to thofe who may have opportunities, it is worthy a trial: none have as yet occurred to me.

In a moſt formidable caſe of pleuriſy, where the uſe of the lancet was judged imprudent, it acted like a charm. In this caſe (a young man aged eighteen), the breathing was more quick, difficult, and anxious than I had ever obſerved it in any inſtance. The countenance was expreſſive of the greateſt diſtreſs. The pulſe was 112 and feeble, with general proſtration of ſtrength. The danger ſeemed imminent, and if he ſurvived the preſent urgent attack, phthiſis as a conſequence was to be dreaded. The pulſe was in a few days reduced to 48, and permanently kept from that to 56. The ſurgeon who attended with me numbered it once at 46. This was the only inſtance that occurred to me, where neither the ſtomach or head were affected under ſo great a reduction.

Mr. Harrold informs me, a youth about nine years of age was ſpeedily cured by the digitalis of a violent convulſive cough, which before baffled all his efforts.

It promiſes to prove permanently ſucceſsful in a caſe of diſtinctly-marked epilepſy. Maſter Tweed, aged fourteen, had been ſubject to frequent returns of epileptic fits from early infancy, the longeſt intervals being three days, and he frequently had three in one day. Every remedy that has been recommended for this diſeaſe, both by regular and irregular practitioners was tried, but in vain. At my requeſt, the fox-glove was given in ſubſtance, made into pills with confect. aromat. under the immediate direction of his father, an eminent ſurgeon at Bocking, in Eſſex. The firſt week after beginning it he had three fits, the laſt on the 12th of December laſt. He has remained perfectly free ſince, and from having almoſt conſtant ſtupor, great languor, bloated, ſwollen countenance, with impaired ſtrength and appetite, his health is perfectly reſtored, and he is become briſk, lively, and active. He was kept under its influence, more or leſs, for upwards of five months.

I have tried it in another caſe ſince, for upwards of a month, but was obliged to diſcontinue it from my ſtock of tincture being expended. It ſeemed to lengthen the intervals from a week to a fortnight, but the fits were more violent. My hopes in this caſe are not very ſanguine; but the fox-glove deſerves farther trials in epilepſy.—The pulſe was preternaturally ſlow in this caſe, being 67 before beginning the tincture, and it may be worthy of remark, that when under its full effects, it was in general quickened. Thirty drops, three times a day, was the utmoſt extent to which it could be carried, though a ſtrong, active young man.

*Some of the moſt remarkable Effects of Fox-glove.*

It is neceſſary for thoſe unexperienced in its uſe to be in poſſeſſion of certain tain *criteria* to direct them in its exhibition, and to enable them to judge how far they may venture to proceed with safety. The most common effects observed from full doses, are vertigo, pain, or throbbing of the forehead, or in the bottom of the orbits; imperfect vision, as if a cloud or mist were passing before the eyes, or as if small spots were moving or dancing in the air; nausea of the most distressing kind, and vomiting; reduction in the frequency of the pulse, or extreme irregularity of it. Sometimes it beats in a slow, uniform, regular manner, and becomes fuller than before. More generally, however, from being slow, it runs on rapidly for a few pulsations, then resumes its former standard, or the finger loses now and then a complete stroke. When slow it is quickened on the slightest bodily exercise. I have never been able to reduce it under fifty in phthisis, without the head and stomach being affected; so that those who expect to succeed in reducing the pulse to any considerable degree without these effects, will be disappointed. In dropsy, where the excitability is not in general so great, this may be done more readily. Some stomachs are extremely susceptible of it, while others bear a full dose without inconvenience. Twenty drops of the tincture sometimes produce immediate but temporary nausea, before the habit has felt its effects. Drowsiness often occurs to a great degree, and from passing restless nights they sleep sound. The intellectual operations are greatly disturbed, for my patients become unfit for any occupation that requires mental exertion. But the most striking effect arising when under its full influence is, to use their own words, "a faintness or sinking at the stomach, as if their life was going from them," different from any thing they ever experienced before. This attracted my attention more than any other, because they all complain of it, and nearly in the same words. When this exists to a very great degree, with constant disposition to fainting, extreme languor, and cold, clammy sweats, it has been carried beyond due bounds, and we ought instantly to desist. The urine, from being high coloured, and depositing a thick sediment, sometimes assumes its natural colour, and is increased in quantity; at other times it is not sensibly changed.

I generally begin with from ten to fifteen drops, three times a day, increasing two drops every second day, until the habit begins to feel its influence. I then desist, and afterwards diminish in the same gradual manner, or augment the dose according to the effect. By these means, the body may, with the greatest safety, be kept under its influence for weeks, and even months.

When there is intense heat and thirst, with flushed cheeks, I direct it to be given in a saline mixture, or draughts; and when these and the ordinary hectic symptoms are subdued, and only weakness remains, tonics are generally rally neceſſary to complete the cure. It is a miſtaken idea that ſtimulants muſt be given in combination with it.

The diſtreſſing nauſea and ſickneſs do not interfere with its ſalutary effects; the immediate relief ſometimes experienced after vomiting, leads me to think they contribute to its ſucceſs; and, from the quantity of bile and viſcid mucus thrown up, without much exertion, the biliary and pancreatic ſecretions ſeemed increaſed by it. The appetite, from being conſiderably impaired, becomes ſometimes very keen, and they can eat heartily during the intervals of nauſea and vomiting. I never knew the bowels violently affected by it in phthiſis, as they frequently are in dropſy; but it keeps them moderately open: nor have I ever obſerved ſalivation or the ſweetiſh taſte of the mouth from it; but this may be owing to my experience being much more limited in conſumption.

In anſwer to the epithets "dangerous, deleterious", and the like, that are continually beſtowed upon it, I think it incumbent upon me to obſerve, that a fatal inſtance never occurred from its uſe, under my care; and I have, I am perſuaded, preſcribed it in upwards of 200 caſes, nor have I ſeen alarming ſymptoms from it, except in two inſtances, where from neglect it was increaſed after the habit was under its full dominion; and I will venture with confidence to affirm, that any ſerious conſequences that have ariſen, or may ariſe, from it, have been, or will be, from inattention to its effects; as it invariably gives full warning of its deleterious qualities. The moſt valuable medicines in our poſſeſſion, or even the moſt ſalutary ſubſtances, will prove injurious, if adminiſtered to exceſs. It is unfair to argue of the uſe of any ſubſtance from the abuſe of it. Opium, tartar emetic, the active preparations of mercury, and many other valuable remedies, are deſtructive poiſons if adminiſtered in undue quantities.

*Mode of Preparation and Exhibition.*

It becomes an object of importance to have a preparation as a general ſtandard which poſſeſſes its virtues unimpaired at all times, and can readily be procured at all ſeaſons, as medical men will then be preſcribing and making obſervations on the ſame remedy, which is really not the caſe at preſent. The infuſion or decoction of the freſh leaves are preferred by Dr. Fowler, but as the plant is not to be had in many diſtricts, even counties, and where it is, as a ſcanty ſupply only is to be procured in winter, with its virtues probably impaired, this preparation is, on theſe grounds, inadmiſſible.

I always uſed the powder in ſubſtance, or the infuſion of the dried leaves in conſumption, as I had done in dropſy, until I heard that Dr. Drake attributed attributed his fuccefs in the two cafes alluded to, to the tincture. I have fince ufed it in feveral cafes, and now give the preference to it, not becaufe I think it poffeffes fuperior virtues to any of the other preparations ufually reforted to, but becaufe the dofe can with more certainty and accuracy be regulated, and it retains its active properties longer, moft probably, for years. I imagined at firft, it acted more readily on the heart, without affecting the ftomach or head, but I am now convinced, this is not the cafe. In dropfy the other preparations merit a decided preference, as they act more readily on the abforbent fyftem, at leaft in taking up ferous fluids.

Having experienced fo many difappointments from the quality of the medicine when I firft began to prefcribe it, I have for fome years cultivated the plant in my garden, whereby I have reafon to believe its virtues are improved: they certainly are not impaired. The leaves have a greener hue, are thicker, more tender and fucculent than thofe growing in the natural ftate. Thefe are often furrounded by weeds and other plants, which deprive them of a fufficient fupply of light, air, and nutriment from the earth, and thereby prevent them from attaining to full perfection. When the ftalks grow thick, ftrong, and tall, with a full fupply of large, thick, green leaves, the plant is in full health and vigour. I begin to gather the leaves when the lower bloffoms are fully expanded, and I have a fecond crop of fmaller ones; but the time of gathering is of little confequence provided they appear green and healthy. They are either dried flowly in the fhade, or quickly in the fun, or near a fire; but they muft not be alternately in the fun and fhade, otherwife they lofe their colour and their virtues. I pafs a packthread through the ftalks, and fufpend them in the fhade, where a current of dry air paffes through them. When thoroughly dried, they are packed clofe in brown paper bags, and fufpended near the kitchen fire. In this manner they will retain their active properties unimpaired for a long time, but I never truft to them for more than one year. When wanted for ufe, no more than will foon be confumed fhould be powdered, as, by frequent expofure to the air, the powder fpeedily becomes an inert fubftance. I wifh particularly to infift on thefe precautions, becaufe few vegetables require greater care in preparing; and becaufe I know many difappointments have arifen from the want of them, which have operated powerfully againft the more general introduction, and confequently the fuccefs of this valuable article. What is fent from London is mixed with the ftalks, and is of no ufe. I have prefcribed it to the extent of feveral grains, without the fmalleft effect, and from feveral failures, I had nearly abandoned, as ufelefs, a medicine, which I have now no hefitation in affirming, is one of the moſt valuable in the whole range of the Materia Medica.

A ſingle grain of the powder prepared as above, the fibres being carefully excluded, is a medium doſe for an adult to begin with, and taken evening and morning, with a little confect. aromat. has frequently evacuated the water in general anaſarca, in forty-eight hours. A grain and half, however, may in general be began with, twice a day, increaſing half a grain a doſe every ſecond day, until ſome effect be obvious. The leaves when dry, ſhould appear green, and have a flavour like ſound freſh hay.

I have tried various proportions of the leaves to ſpirituous menſtrua, for tinctures. The proportions recommended by Dr. Darwin, are an extravagant waſte of the leaves, and of the tincture; for without the ſtrongeſt preſſure, a great portion of the latter is loſt. I have found that of the tincture made according to the following formula, which I beg to ſubmit to the conſideration of the learned College for inſertion in their Pharmacopœa, I have never been able to exceed gtt. xxx. ter. in d.

R/ Folior. digit. purpur. recent. exſiccat. unc. i.
Sp. vin. ten. unc. viij. M.
Digere leni calore per dies ſeptem dien cola.

Or the following, which I ſtill prefer, as having the plant in its perfect ſtate:

R/ Folior. digital. purpur. recent. unc. iv.

Sp. vini. rect. unc. v. M.

Digere dies ſeptem leni calore dien cola.

This makes a beautiful dark-green tincture. I have found by drying the leaves, that they loſe from three-fourths to four-fifths (for they vary much in the degree of humidity they contain) of their weight; ſo that in the above formula I allow three ounces of water contained in the leaves, to the five of pure ſpirit, which is a larger proportion of the ſpirit than is contained in the firſt formula, but nearly the ſame of the leaves. For infuſion dr. ij. of the dried leaves, to unc. viij. of boiling water, ſuffered to remain in a cloſe veſſel till cold, make a very ſtrong infuſion.

Were the College to admit theſe, or any better formulas that may be preſented to them, their ſanction would give more general currency to the fox-glove.

I have for ſome years ſupplied the practitioners with whom I preſcribe this medicine; very few, till lately, at my requeſt, paying attention to its prepapreparation. I have now fome pints of the tinctures made as above, and fome pounds of the leaves in my poffeffion, and though my confumption increafes yearly, fhall be happy in furnifhing any of your numerous readers who may be defirous of giving trial to them, with a portion of either.

I have requefted Mr. Brown, Wholefale Chemift and Druggift, Lothbury, London, to collect a quantity of the leaves of this feafon, and gave him the neceffary directions for making the tinctures, &c.

*Conjectures refpecting its Mode of Action.*

I fhould not, in this little abftract, have entered into any fpeculative opinions on its *modus operandi* on the living fibre, did I not fee theories advanced (on which indications of cure are grounded), that appear to me totally inadmiffible.

That its good effects do not always depend upon its power of diminifhing fecretion, and promoting abforption, as maintained by Drs. Fowler and Drake, is obvious from this, that it is equally, and indeed, more efficacious in cafes where there is no increafe of mucus or pus.

Dr. Drake affirms, " that it has been afcertained, that hectic fever arifes only from the matter of an open ulcer." But however high the authorities that may be adduced in fupport of this opinion, daily obfervation contradicts it.

In the early ftages of confumption it is well known, that the hectic fever is often clearly and diftinctly marked, without any increafed expectoration, and when the tubercles are ftill in their infancy, and confequently before they have fuppurated. Scrophulous enlargements of the mefenteric glands, will, I believe, produce confirmed hectic, and deftroy life, without arriving at maturation. This, if I miftake not, I have repeatedly obferved in children whofe parents, efpecially the mothers, were confumptive, or had fcrophulous affections externally on the body. Females who have furvived two or more pregnancies under phthifis, fometimes produce children that are hectic at birth, and die of marafmus, from mefenteric indurations, in early infancy.

In convulfive coughs its good effects are equally confpicuous, where there is no inordinate fecretion to leffen or abforb.

Nor does its efficacy depend *entirely* on the reduction of the contractions of the heart; for in fome of my patients, the cure was completed without any remarkable diminution of the frequency of the pulfe.

On

On looking over the reports of Miſs Canham's caſe, the pulſe was only once brought to 48, and this and the preceding day, when it was at 52, the ſtomach and whole body were ſo diſordered that ſhe was confined to her bed. The following day the digitalis being diſcontinued, it roſe to 66, and except once that it was 64 and 84, 90 is the loweſt number I obſerve in my notes. Brewſter's never uſed to be reduced lower than 90, and this only once. In both it was originally extremely frequent and ſmall, from 120 to 140.

In a caſe of confirmed phthiſis, with diſtinctly marked ſcrophulous habit, now under my care, which promiſes to do credit to the fox-glove, the pulſe was only 64, prior to his beginning it, which I conſidered as preternaturally ſlow; and it is remarkable, that it has been quickened by it; having been 68, 72, and 76, when examined ſince, nearly at the ſame hour, and under the ſame circumſtances. The head and ſtomach are affected to a great degree by twenty and twenty-two drops of the tincture, three times a day. In this perſon, beſide the ordinary effects, objects ſometimes appear of a green colour that are not ſo in reality.

It is obvious, however, that if the blood can be made to paſs through an organ whoſe excitability is morbidly increaſed in a very high degree, 60 or 50 times in a minute, or even leſs, inſtead of 140 or 120, a very important end will be obtained.

But the theory on which Dr. Drake particularly inſiſts, and on which his curative indications are chiefly built, appears to me liable to inſuperable difficulties.

" It has been lately maintained," ſays he, " by the moſt celebrated phyſioligiſts, among whom Mr. Hunter ſtands foremoſt, that pus is a ſecreted fluid, the conſequence of certain diſeaſed motions of the extremities of the blood-veſſels; it has been likewiſe aſcertained, that hectic fever ariſes only from the matter of an open ulcer; that what is termed laudable pus, when ſecluded from the air is neither capable of creating fever, nor, except by its gravity, can it irritate the parts on which it reſts. When pus, however, is expoſed to atmoſpheric air, it rapidly attracts oxygen, an acid of a peculiar kind is generated, and hectic fever, the effect of the abſorption of aerated matter, is produced. Now as an ulcer of the lungs is perpetually expoſed to a ſtream of air, and of courſe an ichorous poiſon, continually forming by the union of oxygen with ſecreted matter, an important curative proceſs would ſeem to ariſe, from promoting abſorption ſo rapidly from the ſurface of the diſeaſed parts, that the pus ſhall be taken up as ſoon as ſecreted, and conſequently its combination with oxygen prevented." *

That

* " Medical Contributions," p. 480.

That pus was a fecreted fluid I have long believed; that it attracts oxygen, I do not pretend to deny; and that an acid of a peculiar kind may be generated, I take for granted, becaufe it is prefumed the fact has been afcertained by experiment; but that the inferences deduced from thefe premifes are founded in fact, I fee neither evidence nor probability. That hectic arifes from collections of matter in different parts of the body, and more efpecially from vomicæ in the lungs themfelves, without any communication with the external air; and that the fluid thus formed, is frequently not a laudable pus, but a thin offenfive ichor, it is only neceffary for me to call inftances to the recollection of every medical man of obfervation. It is ftill more difficult to admit that the hectic fever arifes from the abforption of the peculiar acid, or ichorous poifon, generated by the union of oxygen with pus. If this were the cafe, the thick bland matter fecreted from every wound or ulcer, when expofed to a ftream of air, would become an ichorous poifon, and be productive of the effects mentioned by Dr. Drake; but that this does not in reality happen, daily obfervation fufficiently evinces. Even in the lungs themfelves, when from pleurify or pneumonic inflammation, fuppuration happens in a conftitution previoufly found, good, thick pus, without any fœtor, is fometimes formed; and we continually obferve both in the lungs and other parts, the acrid offenfive ichor of ulcerated furfaces, converted into a copious fecretion of what is termed laudable pus; whereas, while any is continued to be fecreted, being equally expofed to the air, it ought ftill, acording to this theory, to be a thin ichor. It appears to me obvious, that caufe is here confounded with effect, and effect with caufe; and that the ill-conditioned matter thus poured out, is the immediate confequence of the highly morbid ftate of the veffels fecreting it, as well as of the whole body, and not of any fubfequent changes it undergoes; and that it will be vain to attempt to improve the one until the other be corrected. A wound or ulcer in any part of the body under confirmed hectic, would moft probably difcharge a thin offenfive ichor, and every judicious furgeon would endeavour to correct the morbid condition of the habit which occafioned it, before he expected to fucceed in healing it. Good pus he would look for as a neceffary confequence; nor would he under any circumftances, endeavour to promote its abforption.

A peculiar delicacy of conftitution, with a high degree of fufceptibility, both of body and mind, are mentioned by all authors as characterifing the true genuine phthifical habit. Hence the flighteft caufes often produce the moft powerful impreffions. The circulation is accelerated by ftimuli, which in in others produce no more than the healthy actions. A cough, pain of the side, or some local affection, supervene to every little exposure to cold, or transition of temperature; and a cough once arising in such habits, without extreme caution, will soon lay the foundation of the disease. The inflammation of the mucus membrane and bronchial glands, at first trifling, proves a constant source of irritation to the whole habit. The blood will be urged with increased velocity through every part; and the muscles subservient to respiration being thrown into repeated convulsive actions, the pulmonary system will more especially suffer from the effects of congestion, distention, increased action, and impetus. Indurations of the glands, or tubercles, will be formed, which, by degrees, run through the progressive stages of inflammation and suppuration; and as the pus thus generated cannot be expected to be of a good bland kind, it proves by its acrid qualities, like oil to a lamp, additional fuel to the flame which gave birth to it. I do not mean to infer, that this is always the progress observed by the disease, but believe it is often so of the most dangerous and fatal kind; and whatever way it originates, the effect will be the same. To lessen or remove this morbid excitability should be the grand object of our art to attain, if we expect to be successful in preventing, or when once formed, in permanently removing consumption. But this is extremely difficult—often impossible. Not only the diet, dress, peculiar habits and occupations, but the climate itself, must be changed, before so important an end can be gained. When tubercles are once formed to any considerable extent or degree, especially in scrophulous habits, where the glandulous system is in a state of continual predisposition to inflammation of the most hopeless kind, it is often too late. The fox-glove, however, seems to me to possess powers over every other remedy yet known, in correcting this morbid condition of the whole frame, and the train of morbid phenomena resulting from it. And it is to these I have been disposed to attribute, in a great degree, its salutary effects in this deplorable malady. If it frequently possess such controul over the heart as to reduce its contractions from 140 and 120 to 50 in a minute; if it allay, as it does in a most extraordinary manner, the cough and irritation of the lungs, and indeed of every part, the advantages thence resulting will be incalculable. The vessels of the diseased lungs will be placed in a condition of secreting bland, healthy fluids, every organ in a state of performing its healthy functions, and thus the unison and harmony which constitute the healthy standard will be established throughout the body.

As in other cases the lymphatics are the artificers, or active agents employed by Nature in removing inorganic matter, either solid or fluid, that that has no other ready outlet; ſo in this, what cannot readily be expecto-rated may be taken up by them and eliminated through the various emunc-tories. But whether they poſſeſs the extenſive powers attributed to them by Dr. Fowler, and which they certainly do in abſorbing ſerous fluids, appears to me extremely doubtful.

---

